# The Selection and Use of Outcome Measures in Palliative and End-of-Life Care Research: The MORECare International Consensus Workshop

**DOI:** 10.1016/j.jpainsymman.2013.01.010

**Published:** 2013-12

**Authors:** Catherine J. Evans, Hamid Benalia, Nancy J. Preston, Gunn Grande, Marjolein Gysels, Vicky Short, Barbara A. Daveson, Claudia Bausewein, Chris Todd, Irene J. Higginson, MORECare

**Affiliations:** a Department of Palliative Care, Policy and Rehabilitation, Cicely Saunders Institute, King's College London, London, United Kingdom; b School of Nursing, Midwifery & Social Work, University of Manchester, Manchester, United Kingdom; c International Observatory on End of Life Care and Faculty of Health & Medicine, Lancaster University, Lancaster, United Kingdom; d Centre for Social Science and Global Health, University of Amsterdam, Amsterdam, The Netherlands; e Interdisciplinary Centre for Palliative Medicine, Munich University Hospital, Munich, Germany

**Keywords:** Outcome assessment, evaluation studies, research design, palliative care, consensus

## Abstract

**Context:**

A major barrier to widening and sustaining palliative care service provision is the requirement for better selection and use of outcome measures. Service commissioning is increasingly based on patient, carer, and service outcomes as opposed to service activity.

**Objectives:**

To generate recommendations and consensus for research in palliative and end-of-life care on the properties of the best outcome measures, enhancing the validity of proxy-reported data and optimal data collection time points.

**Methods:**

An international expert “workshop” was convened and an online consensus survey was undertaken using the MORECare Transparent Expert Consultation to generate recommendations and level of agreement. We focused on three areas: 1) measurement properties, 2) use of proxies, and 3) measurement timing. Data analysis comprised descriptive analysis of aggregate scores and collation of narrative comments.

**Results:**

There were 31 workshop attendees; 29 recommendations were included in the online survey, completed by 28 experts. The top three recommendations by area were the following: 1) the properties of the best outcome measures are responsive to change over time and capture clinically important data, 2) to enhance the validity of proxy data requires clear and specific guidelines to aid lay individuals' and/or professionals' completion of proxy measures, and 3) data collection time points need clear identification to establish a baseline.

**Conclusion:**

Outcome measurement in palliative and end-of-life care requires the use of psychometrically robust measures that are clinically responsive, with defined data collection time points to establish a baseline and clear administration guidelines to complete proxy measures. To further the field requires clinical imperatives to more closely inform recommendations on outcome measurement.

## Introduction

Widening access to palliative and end-of-life (EOL) care services is advocated with corroboration of patient^1^ and carer benefits,^2^ greater potential for health service cost savings,^3,4^ and increasing demand with an aging population.^5^ A major barrier is the requirement for better selection and use of outcome measures to demonstrate the effectiveness of services. Commissioners of health and social care services increasingly require service providers to use patient, carer, and service outcomes to demonstrate a service's safety, effectiveness, and quality as opposed to detailing service activity.^6,7^ In research and clinical practice, a multitude of measures are used, and frequently these measures are not validated with palliative care populations.^8^ This hampers meta-analyses, limits responsiveness to change in outcome, evaluating service effectiveness, and developing the evidence base to inform best practice.^9^

Trials and nonrandomized designs in palliative and EOL care are often compromised by the use of untested outcome measures,^10,11^ measures not developed for palliative care populations,^12^ uncertainty as to the best measurement time points,^13^ and the use of measures for symptom change with less use of measures encompassing the multiple domains of palliative care (e.g., Palliative care Outcome Scale [POS], Edmonton Symptom Assessment System).^12^ These limitations are not unique to palliative and EOL care. The COnsensus-based Standards for the selection of health Measurement INstruments is a comprehensive checklist for assessing the quality of the measurement properties of health status questionnaires. The checklist was developed in response to the rapid increase in health status questionnaires and the need for quality criteria to compare measures in systematic reviews, identify shortcomings, and design studies validating measures.^14,15^

Palliative and EOL care involves people with increasing debility associated with advancing disease and has a broad mandate of care provision, with intended outcomes of improving quality of life for patients and their caregivers.^16^ The nature of palliative and EOL care requires measurement properties that accommodate the following: the multiple domains of palliative care; to change over time and increasing levels of fatigue; the use of proxies, particularly when individuals are near to death; and timing to detect change and monitor sustainment of change. These challenges are well reported, and international advancements to address them are evident: projects such as the Palliative Care Outcomes Collaboration in Australia that uses national standardized assessments of palliative care outcomes^17,18^ and a European collaboration, entitled PRISMA, focused on promoting best practice in the measurement of EOL care (Preferred Reporting Items for Systematic Reviews and Meta-Analyses)^11^ working with a European Association of Palliative Care (EAPC) Taskforce on patient-reported outcome measures (PROMs) in palliative care to further the work^19,20^ and provide guidance on selecting and using measures.^21^ Internationally, there is increasing use of multi-domain palliative care measurement tools in research and clinical practice (e.g., POS and Support Team Assessment Schedule [STAS]).^22^

No work, however, has considered specific recommendations for the use and selection of outcome measures in trials and nonrandomized studies of palliative and EOL care interventions. This article reports on an international outcome measurement workshop and online consensus survey that aimed to identify agreed best practice on selecting and using outcome measures. The work is part of the Methods Of Researching End of life Care (MORECare) project, developing evidence-based guidance on the design and conduct of evaluative research on palliative and EOL care. MORECare integrated data from three systematic reviews^23–25^ and five workshops^26,27^ to generate a statement (the MORECare statement) that sets clear standards on good research practice in evaluating services and treatments in palliative and EOL care.^28^ This paper reports on the outcome measurement workshop.

## Methods

### Setting

An invitation-only workshop was held as a joint initiative between the MORECare and PRISMA projects at the 2011 EAPC conference to further the European work undertaken by PRISMA^11^ and the EAPC Taskforce on PROMs in palliative care.^19^

### Participants

Holding the workshop at an international conference intended to enable participation by international experts in palliative care research, service provision, and policy. Participants were identified from the EAPC online conference program and considered experts from their publications. Fifty-nine individuals were identified and received a personal e-mail invitation to attend the workshop and a single reminder e-mail.

### Design

**Fig. 1:**
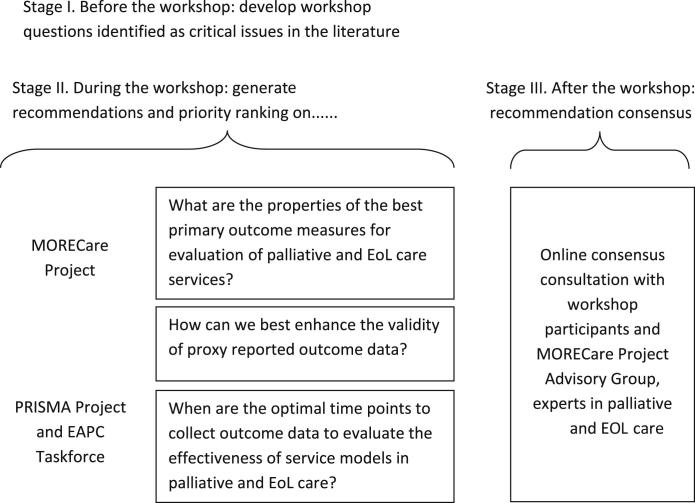
Flow diagram of the study design.

We used the MORECare Transparent Expert Consultation method, which incorporates consensus methods of nominal group and consensus survey.^29^ The Transparent Expert Consultation comprised a staged process: Stage I—identifying critical issues from the literature; Stage II—a workshop with experts on the issues using nominal group technique to generate recommendations; and Stage III—an online consensus survey to prioritize and identify areas of contention/uncertainty (Fig. 1).

#### Stage I: Identifying Critical Issues

Critical issues on selecting and using outcome measures in palliative and EOL care were identified from systematic reviews of intervention studies^12^ and outcome measurement.^30^ The workshop and consensus survey focused on three critical questions:

1. What are the properties of the best primary outcome measures for evaluation of palliative and EOL care services?
2. How can we best enhance the validity of proxy-reported outcome data?
3. When are the optimal time points to collect outcome data to evaluate the effectiveness of service models in palliative and EOL care?

#### Stage II: Workshop

Participants received a pre-workshop briefing pack detailing the aim, critical questions, and workshop format. The workshop format comprised presentations on the MORECare project and the project's interface with the PRISMA/EAPC Taskforce, followed by three structured groups of seven to 13 participants each focused on one of the critical questions. The group structure used a modified nominal group facilitated and scribed by members of the MORECare and PRISMA projects. The facilitators guided participants through a structured process of: 1) brief discussion, 2) individual writing of recommendations and ranking, and 3) individuals in turn reading out their highest ranked recommendations until individual lists were exhausted (or time exceeded).^31^ The scribes wrote the recommendations and ranking on flip-charts, and each small group discussed and agreed on the final priority order, which they presented and discussed with the whole group. Participants listed and ranked recommendations from one to five (highest to lowest) on structured A4 sheets detailing the respective group question, the ranking scale and boxes to list recommendations, rank, and detail rationale.

#### Stage III: Consensus Survey

Recommendations were posted online to the workshop participants (excluding the facilitators and scribes) and members of the MORECare Project Advisory Group. Participants received a personalized e-mail invitation and reminder after two weeks. The online participants ranked, from one to nine (strongly disagree to strongly agree), the extent they agreed with a recommendation and used free-text spaces to comment on each recommendation.

### Data Analysis

Individual recommendations and ranking were entered into Excel spreadsheets with assigned participant identification numbers. Flip-chart records and the scribes' notes were typed. Three researchers (C.J.E., H.B., M.G.) drew on qualitative analysis to identify themes in the recommendations and assign codes^32^ and collate typed data. Coded recommendations were rearranged by theme in Excel spread sheets. Duplicates were combined and recommendations arranged by priority ranking. The recommendations retained participants' original language, with amendments to enhance clarity. A third researcher (I.J.H.) reviewed the analysis and proposed recommendations. After combining for duplicates, we included all recommendations in the online survey that participants had ranked three and above and/or participants' listed recommendations formed a prominent theme. The MORECare research team reviewed and agreed on the recommendations for the online survey.

Analysis of scaled data followed conventional rules of descriptive statistics (frequencies and medians) and plots (box and whisker plots of interquartile ranges to analyze^33^ and interpret levels of agreement [Table 1]).^34^ Narrative comments were collated by recommendation, themes identified, and rearranged in Excel sheets to enable comparison.^35^ The analysis of comments intended to aid understanding and provide illustrative examples of the issues raised by the proposed recommendations.^36^

### Ethics

The study was approved by the University of Manchester Research Ethics Committee (reference number: 10328).

## Results

### Participants

Thirty-one international and national experts attended the workshop comprising senior academics, policy makers, and clinicians, representing countries from Europe (e.g., Norway, Belgium, U.K., Italy, Germany), North America, and Australia. All held a title of professor or doctor. Twenty-eight experts completed the online survey.

### Recommendations and Levels of Agreement

**Fig. 2:**
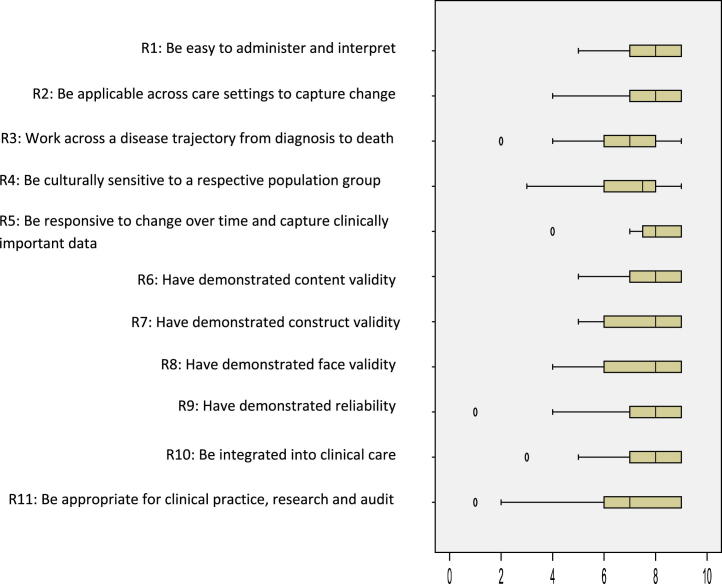
Properties of the best primary outcome measures in evaluations of palliative and EOL care. Box and whisker plot of the interquartile ranges and medians of level of agreement for the 11 recommendations (box: 25th and 75th percentiles; whiskers: minimum and maximum).

**Fig. 3:**
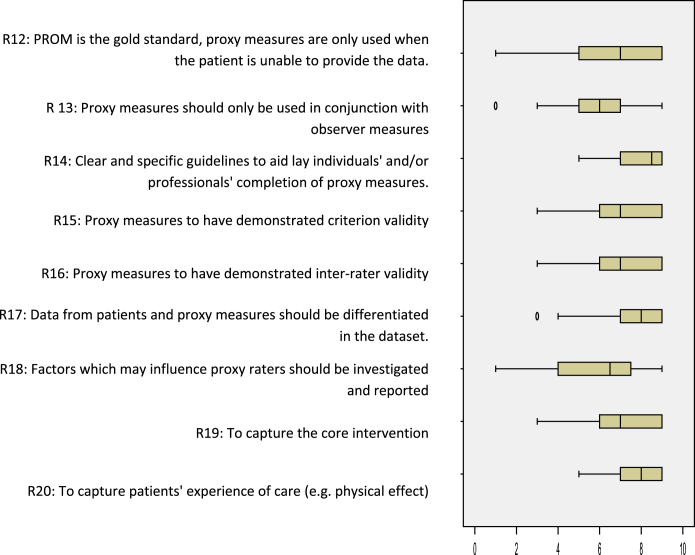
Enhancing the validity of proxy data in evaluations of palliative and EOL care. Box and whisker plot of the interquartile ranges and medians of level of agreement for the nine measurement recommendations (box: 25th and 75th percentiles; whiskers: minimum and maximum).

**Fig. 4:**
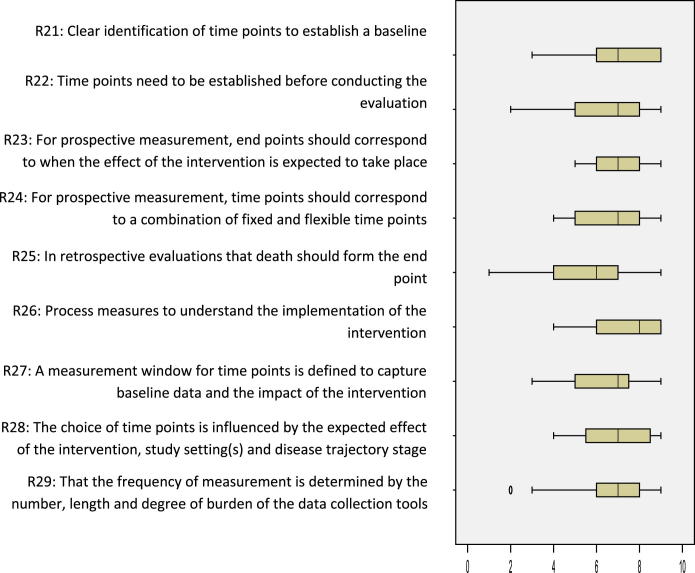
Data collection time points in evaluations of palliative and EOL care. Box and whisker plot of the interquartile ranges and medians of level of agreement for the nine recommendations (box: 25th and 75th percentiles; whiskers: minimum and maximum).

The workshop generated 155 individual recommendations; 29 recommendations were included in the online consensus survey after combining duplicates and completing analysis. The recommendations included in the online survey were either generic describing good practice in choosing and administering outcome measures in effectiveness research or focused on specific challenges in research on palliative and EOL care (Table 2). Overall, the median levels of agreement for the 29 recommendations showed that most recommendations (*n* = 26) were indicated with medians ≥7; none were not indicated (medians ≤3) (Table 2). However, the levels of agreement across the three areas varied. The recommendations on: 1) the properties of outcome measures showed mainly strict agreement that the recommendations were indicated (*n* = 6/11 [Fig. 2]), 2) enhancing proxy data showed mainly broad agreement (*n* = 6/9 [Fig. 3]), and 3) data collection time points all showed broad agreement (*n* = 9 [Fig. 4]). Recommendations in which the level of agreement was equivocal or broad were considered as uncertain or contentious. Qualitative analysis of the workshop discussions and the online survey free-text comments informed understanding on these areas. We present quotes to illustrate these debates.

### Properties of the Best Outcome Measurements

Properties of the best primary outcome measures formed 11 recommendations (Fig. 2; Table 2). Overall, there was strict agreement that the properties of the best outcome measures required valid measures that were responsive to change over time, captured clinically relevant data, and were easy to administer across care settings (Recommendations 1, 2, 5, 6, 9, 10). The text data indicated that measures required flexibility in the administration of the measure (e.g., PROMs, observation) to ensure responsiveness to patients' fluctuating capacity and increasing fatigue, notably in the last days and hours of life. An area of less certainty was the recommendation that a measure was appropriate for clinical practice, research, and audit, with broad agreement indicated but with the widest spread (Recommendation 11) (Fig. 2).

Uncertainty centered on accommodating competing priorities for practice, research, and audit, summarized by a survey respondent as:

It is impossible to serve all 3 masters optimally. They need to be optimized for one or perhaps two goals. (ID OMs 6)

Measures in research required greater detail than the comparatively brief measures required in clinical practice. However, there was agreement on the importance of research capturing clinically relevant data (Recommendation 5) that could be integrated into clinical care (Recommendation 10). A way forward discussed in the workshop and in the online survey comments was the use of “core outcomes” with additions depending on the context, as a survey respondent stated:

… core outcomes (for all settings) and others setting-specific, this will give a more accurate approach, for the process and for specific settings. (ID OMs 12)

### Enhancing the Validity of Proxy-Reported Outcome Data

Greater uncertainty surrounded the seven draft recommendations on enhancing the validity of proxy-reported data, with wide variance in degrees of agreement (Fig. 3). There was strict agreement that to enhance proxy measures required clear specific guidelines to aid completion, the differentiation of patient and proxy data in datasets and that proxy measures captured patients' experiences of care (Recommendations 14, 17, 20) (Table 2).

Two recommendations were equivocal and concerned increasing the reliability of proxy measures and understanding bias (Recommendations 13 and 18). Recommendation 13 that proxy measures should only be used in conjunction with observer measures was viewed as useful in the absence of PROMs data. However, uncertainty surrounded the validity of using such measures in conjunction and the best method of statistical analysis. Best research practice meant using valid measures, but further research was required on the contribution of using measures in conjunction to enhance reliability. There was agreement that Recommendation 18 was indicated on the importance of investigating factors that may influence proxy raters to reduce bias. However, participants' comments questioned the feasibility of this in research practice because of the complexity in collecting and interpreting such data.

The main area of contention was the recommendation on the precedence of PROMs over proxy measures, indicated by the wide spread (Recommendation 12) (Fig. 3). The survey participants' comments indicated that using PROMs data alone negated that palliative care encompassed both patients and their families, would fail to capture data in the last days of life from, for example, observation, and the relevance of clinical data, for example, medication use. Survey participants advocated the requirement for multiple measures in research on palliative care. The workshop delegates advocated that proxy measures were “second choice” over PROMs, but proxy data were “indispensable” in palliative care because of patients' deteriorating capacity and increasing fatigue.

### Optimal Data Collection Time Points in Clinical Trials and Evaluations of Interventions

There was broad agreement that the eight recommendations on time points were indicated (Fig. 4). The wide distribution and no strict agreement on the recommendations suggested uncertainty/contention. The recommendation of death as the endpoint in retrospective data analysis was equivocal (Recommendation 25). The online survey comments revealed that death as an endpoint was considered too narrow as it failed to capture the holistic nature of palliative care, which encompasses carers and family members across the disease trajectory and into bereavement. However, death could form an important endpoint in retrospective data analysis depending on the objectives of the study, as one workshop participant stated:

Use death as [an] endpoint and count burden from this – as time or referral/first assessment may vary so greatly. (ID G2.015)

Although the workshop participants used the term endpoint, this caused confusion in the online survey, and alternative terms of anchor point or starting point were proposed. The confusion likely contributed to wide range of scores and finding of an equivocal recommendation.

## Discussion

Research on palliative and EOL care is beset by well-reported challenges rooted in involving individuals with advanced illness in research and their families, the multiple domains of palliative care provided across health and social care, and the intention to improve quality of life in the face of deterioration.^9,10,12,37^ These challenges have hampered the use and selection of outcome measures in trials and nonrandomized studies on palliative and EOL care services.^38,39^ We critically discuss three main areas to further this field of research: priority measurement properties, incorporating proxy data, and identifying time points.

### Measurement Properties

The findings corroborate work undertaken by the PRISMA project that research on palliative care requires the use of short, brief outcome measures that accommodate patients' increasing levels of fatigue and minimize burden.^8,20,40^ This does not equate to “easy” to measure aspects of care, but areas important to patients and families that encompass desired benefit from a service or intervention.^41^ The challenge is to use measures that capture the multiple domains of palliative care, but in a format that is short and easy to interpret. Our findings support the requirement for measures used in palliative care to be applicable for both clinical practice and research.^11,41^ Work in Australia has demonstrated the feasibility of collecting national palliative care outcome data across specialist services using defined measurement tools incorporated in routine practice and the potential for national benchmarking for care quality and outcomes.^17^ However, our findings and the PRISMA work show that there are competing priorities between the requirements of a measure used in clinical practice and research.^40^ Time pressure in clinical practice necessitates the use of short measures (e.g., six to 10 items)^8^ that are easy to administer and interpret to inform clinical decisions. In research evaluating the effectiveness of an intervention or service, the measure(s) used needs to demonstrate the degree of change over time, not simply the presence or absence of, for example, a symptom. Both clinical and research settings, however, require the use of measures with validated psychometric properties to ensure scientific rigor and enhance clinical decision making.^40^ To accommodate these competing priorities (and similarities) of our findings, the PRISMA work suggests the use of core measures for clinical and research practice and further assessment of specific areas for research when indicated.^8^ Mularski et al.^42^ propose, from consensus work, that evaluation studies use an outcomes measurement strategy that encompasses three outcome categories: condition specific, patient outcomes, and family/caregiver outcomes. Measures such as POS (http://pos-pal.org),^43^ the Edmonton Symptom Assessment System,^44^ and Memorial Symptom Assessment Scale^45^ are examples of measures commonly used in both palliative care clinical practice and research,^41^ are validated with palliative care populations, and are short to administer and multidimensional.

Palliative care is provided internationally across health and social care settings and to patients with cancer and increasingly to those with nonmalignant illness. Our data indicate the requirement for measures to be culturally sensitive and be applicable across care settings to capture change in outcomes, particularly for patients who move between care settings, for example, at home, inpatient hospital, or care home. The development of existing measures is apparent, notably POS, with work to increase cultural sensitivity for countries both in Africa^46^ and Europe,^47^ and use with disease groups other than cancer.^22^ Further validation work of palliative care measures like POS, however, is required for specific patient groups, notably patients with dementia^48,49^ and for social care settings (e.g., care homes).^50^

### Incorporating Proxy Data

Our data showed the clinical complexity for palliative care in using PROMs as the “gold standard” in research. The complexity of palliative care service provision to meet diverse care needs of patients and families necessitates data collection from multiple sources. It is an essential requirement in research on palliative and EOL care to expect and plan for missing data because of deterioration.^27,42^ Proxy-reported outcome data are integral sources in palliative care, particularly at points of a patient's deterioration or unstable symptoms when physical and/or mental debility may prevent use of a PROM. At these points, patients likely experience increasing symptom distress, points at which it is imperative to understand the effectiveness of the care and treatment provided. PROMs, such as POS and Memorial Symptom Assessment Scale, are validated for use with a proxy.^43,49,51^

However, the extent a proxy report reflects a patient response depends on the degree of agreement and the direction of agreement (e.g., proxies report high ratings when patients report low) between the proxy and patient; the greater the level of agreement, the greater the confidence in including proxy reports.^51^ Studies in palliative care have shown wide variance in agreement responses depending in particular on the variable measured and the patient-proxy dyad.^51–53^ Studies, for example, report high agreement for observable patient variables (e.g., ability to transfer) and moderate to low for psychological domains and subjective patient experiences (e.g., quality of life).^53,54^ In patient and caregiver dyads, higher agreement is reported than in patients and health care provider dyads,^51^ but over time, similar reporting is seen as practitioners' knowledge of their patients increases.^53^ Our findings correlate with studies on proxy and patient reporting that to further the inclusion of proxy reports requires greater understanding of the factors that influence reporting to improve reliability (e.g., degree of neutrality, knowledge of patient), further development of proxy measures to capture patients' subjective experiences (e.g., quality of life), and ways to enhance statistical analysis.^51–54^

### Identifying Time Points

Identifying optimal time points intends to maximize the number of patients available at consecutive points of measurement to demonstrate the effectiveness of the intervention.^13^ Our data show that best practice to identify time points was the area of most uncertainty, with broad or equivocal agreement surrounding the recommendations posed. Identifying time points in research on palliative and EOL care is a prominent methodological challenge rooted in minimizing patient burden, imprecision in identifying the state of “dying,” and attrition.^13,34^ In particular, attrition from increasing debility minimizes data collection for individuals with the greatest symptom burden and compromises a study's internal validity with overrepresentation of the “healthiest” patients.^55^ Hence, careful planning of time points is crucial.

Our data show broad agreement for the recommendations on establishing time points before the evaluation, and the frequency of measurement is determined by the degree of burden of the data collection tools. These recommendations are likely too narrow, hence the wide spread of responses. The timing and frequency of measurements is embedded within a conceptual framework for the intervention. The conceptual framework is developed from the research question and detailed understanding of the intended intervention outcome/benefit, the expected response pattern (e.g., two weeks post-consultation with the palliative care clinician), and the phase of illness (e.g., stable disease, deteriorating, dying phase).^18,56,57^

The recommendation of death as an endpoint in retrospective evaluations was equivocal. This likely reflects misunderstanding of the term “endpoint.” The use of anchor point from which data are included may have increased clarity. Retrospective evaluations in palliative care are important study designs. Prospective designs are hampered by imprecision in the identification of the state of dying, particularly in measures of quality of life, which often deteriorate in the last days of life.^13^ A way to accommodate this is prospective observations and data analysis that uses both prospective analysis from the baseline and retrospective analysis from the last observation before death.^58^ Death is a fixed anchor point for the whole sample and provides more reliable data on the clinical outcomes over time.^59^

### Limitations

We undertook the consensus workshop at an international conference in palliative care. This enabled participation by international experts but limited invitations to those attending the conference and the time available, particularly for discussion. The use of clear questions and a structured process to generate the recommendations was intended to maximize the time. We limited the online consensus to a single round to enhance the response rate. This prevented wider consultation on the survey comments that detailed reasons for scoring as well as measures of central tendency and dispersion. There is no agreement on the best method for mathematical aggregation in consensus surveys.^29^ We used an implicit approach of medians and measure of dispersion^29^ and the Jones and Hunter established method for interpreting scaled data.^30^

## Conclusions

To enable the wide application and interpretation of outcome measures in palliative and EOL care, the measurement properties need to be validated for this population, capture multidimensional components, and be broadly applicable across health systems, populations, disease trajectories (including post-death for bereaved carers), languages, and cultures. The best measures have essential properties of simplicity of use and good interpretability, the ability to measure clinically important change, and reporting by a patient or proxy informant. To further the field requires clinical imperatives to more closely inform recommendations on outcome measurement.

## Figures and Tables

**Table 1:** Recommendation Agreement: Interpretation of Median Regions and IQR^34^

Median Regions and IQR	Interpretation
7–9	Recommendations are indicated
4–6	Recommendations are equivocal
1–3	Recommendations not indicated
IQR in *one* region	Strict agreement for recommendation
IQR in *any* three-point region	Broad agreement for recommendation

IQR = interquartile range.

**Table 2:** Recommendations on Selecting and Using Outcome Measures by Area and Level of Agreement

No.	Area 1: Properties of the Best Primary Outcome Measures in Evaluations of Palliative and EOL Care Should …	Median (IQR)
	**Strict agreement that recommendation is indicated**	
1	Be easy to administer and interpret (e.g., short and low level of complexity).	8 (7–9)
2	Be applicable across care settings to capture change in outcomes by location (e.g., patient's home, hospital, hospice).	8 (7–9)
5	Be responsive to change over time and capture clinically important data.	8 (7.5–8)
6	Have demonstrated content validity.	8 (7–9)
9	Have demonstrated reliability.	8 (7–9)
10	Be integrated into clinical care.	8 (7–9)
	**Broad agreement that recommendation is indicated**	
3	Work across a disease trajectory from diagnosis to death.	7 (6–8)
4	Be culturally sensitive to a respective population group (e.g., linguistically and culturally).	7.5 (6–8)
7	Have demonstrated construct validity.	8 (6–9)
8	Have demonstrated face validity.	8 (6–9)
11	Be appropriate for clinical practice, research, and audit uses.	7 (6–9)

No.	Area 2: Enhancing the Validity of Proxy Data in Evaluations of Palliative and EOL Care Services Requires …	Median (IQR)
	**Strict agreement that recommendation is indicated**	
14	Clear and specific guidelines to aid lay individuals' and/or professionals' completion of proxy measures.	8.5 (7–9)
20	Capture of patients' experiences of care (e.g., emotional effect, physical effect).	8 (7–9)
17	That data from patients and proxy measures should be differentiated in the dataset.	8 (7–9)
	**Broad agreement that recommendation is indicated**	
12	PROMs as the gold standard in evaluations of palliative and EOL care services; proxy measures should be used only when the patient concerned is unable to provide the data.	7 (5–9)
15	Proxy measures to have demonstrated criterion validity.	7 (6–9)
16	Proxy measures to have demonstrated interrater validity.	
19	Capture of the core intervention.	7 (6–9)
	**Broad agreement that recommendation is equivocal**	
13	That to increase reliability in evaluations of palliative and EOL care services, proxy measures should only be used in conjunction with observer measures.	6 (5–7)
18	Factors that may influence proxy raters to be investigated and reported (e.g., degree of emotional involvement or neutrality).	6.5 (4–7.3)

No.	Area 3: Data Collection Time Points in Evaluations of Palliative and EOL Care Require …	Median (IQR)
	**Broad agreement that recommendation is indicated**	
21	Clear identification of time points to establish a baseline.	7 (6–9)
22	Time points in evaluations on palliative and EOL care services need to be established before conducting the evaluation.	7 (5–8)
23	That when prospective measurement is used, endpoints should correspond to when the effect of the intervention is expected to take place.	7 (6–8)
24	That when prospective measurement is used, time points should correspond to a combination of fixed and flexible time points (e.g., a flexible time point may be triggered when a threshold is reached).	7 (6–8)
26	Process measures to understand the implementation of the intervention.	8 (6–9)
27	A measurement window for time points is defined to capture baseline data and the impact of the intervention.	7 (5–7.3)
28	The choice of time points is influenced by the expected effect of the intervention, the study setting(s) (e.g., patient's home, hospital) and the disease trajectory and stage.	7 (5.8–8.3)
29	That the frequency of measurement is determined by the number, length, and degree of burden of the data collection tools.	7 (6–8)
	**Broad agreement that recommendation is equivocal**	
25	In retrospective evaluations that death should form the endpoint.	6 (4–7)

EOL = end of life; IQR = interquartile range; PROMs = patient-reported outcome measures.
